# Comparative Genome Analysis of Wheat Blue Dwarf Phytoplasma, an Obligate Pathogen That Causes Wheat Blue Dwarf Disease in China

**DOI:** 10.1371/journal.pone.0096436

**Published:** 2014-05-05

**Authors:** Wang Chen, Yan Li, Qiang Wang, Nan Wang, Yunfeng Wu

**Affiliations:** State Key Laboratory of Crop Stress Biology for Arid Areas, Key Laboratory of Crop Pest Integrated Pest Management on Crop in Northwestern Loess Plateau, Ministry of Agriculture, College of Plant Protection, Northwest A&F University, Yangling, Shaanxi, China; Agriculture and Agri-Food Canada, Canada

## Abstract

Wheat blue dwarf (WBD) disease is an important disease that has caused heavy losses in wheat production in northwestern China. This disease is caused by WBD phytoplasma, which is transmitted by *Psammotettix striatus*. Until now, no genome information about WBD phytoplasma has been published, seriously restricting research on this obligate pathogen. In this paper, we report a new sequencing and assembling strategy for phytoplasma genome projects. This strategy involves differential centrifugation, pulsed-field gel electrophoresis, whole genome amplification, shotgun sequencing, *de novo* assembly, screening of contigs from phytoplasma and the connection of phytoplasma contigs. Using this scheme, the WBD phytoplasma draft genome was obtained. It was comprised of six contigs with a total size of 611,462 bp, covering ∼94% of the chromosome. Five-hundred-twenty-five protein-coding genes, two operons for rRNA genes and 32 tRNA genes were identified. Comparative genome analyses between WBD phytoplasma and other phytoplasmas were subsequently carried out. The results showed that extensive arrangements and inversions existed among the WBD, OY-M and AY-WB phytoplasma genomes. Most protein-coding genes in WBD phytoplasma were found to be homologous to genes from other phytoplasmas; only 22 WBD-specific genes were identified. KEGG pathway analysis indicated that WBD phytoplasma had strongly reduced metabolic capabilities. However, 46 transporters were identified, which were involved with dipeptides/oligopeptides, spermidine/putrescine, cobalt and Mn/Zn transport, and so on. A total of 37 secreted proteins were encoded in the WBD phytoplasma chromosome and plasmids. Of these, three secreted proteins were similar to the reported phytoplasma virulence factors TENGU, SAP11 and SAP54. In addition, WBD phytoplasma possessed several proteins that were predicted to play a role in its adaptation to diverse environments. These results will provide clues for research on the pathogenic mechanisms of WBD phytoplasma and will also provide a perspective about the genome sequencing of other phytoplasmas and obligate organisms.

## Introduction

Phytoplasmas are wall-less bacteria and members of the class *Mollicutes*. They are naturally transmitted by phloem-feeding insects of the order Hemiptera, and they cause diseases in several hundred economically important plants [1]. As obligate parasites, they live strictly within insects and plant phloem [2]. Until now, researchers have been unable to culture them *in vitro*, making it difficult to carry out experimental studies on these pathogens.

The exploration of the genome sequence has an important role in the study of phytoplasmas. However, the collection of sufficiently high-quality phytoplasma genomic DNA poses a challenge to phytoplasma genome sequencing. To date, only five complete phytoplasma genomes have been sequenced. These are strains of onion yellows M (OY-M) and aster yellows witches'-broom (AY-WB), which belong to ribosomal subgroups 16Sr IB and IA of ‘*Candidatus* (*Ca.*) Phytoplasma (P.) asteris’, respectively, Australian isolate and strawberry lethal yellows isolate of ‘*Ca*. P. australiense’, and a strain AT of ‘*Ca*. P. mali’ [3]–[7]. In addition to these five complete phytoplasma genomes, five phytoplasma draft genomes have also been published. These draft genomes are for four 16Sr III phytoplasmas (Italian Clover Phyllody phytoplasma strain MA, Vaccinium Witches' Broom phytoplasma, Milkweed Witches' Broom phytoplasma and Poinsettia branch-inducing phytoplasma strain JR = PoiBII [8]) and one 16Sr II phytoplasma (Peanut Witches' Broom phytoplasma (PnWB) strain NTU2011 [9]).

Wheat blue dwarf (WBD) phytoplasma is the causative agent of WBD disease, which is the most economically important phytoplasma disease affecting winter wheat in arid and semiarid areas of northwestern China, including the Shaanxi, Shanxi and Gansu provinces. Since the 1960s, WBD disease outbreaks have occurred more than 10 times in the Shaanxi province, causing a loss in wheat yield of approximately 50,000 metric tons per epidemic year [10]. WBD phytoplasma is transmitted by *Psammotettix striatus*. The main symptoms of WBD disease are dwarfism and yellow leaf tips. In most cases, the flowers of WBD phytoplasma-infected wheat are sterile, and no grains are produced. In addition, in some susceptible wheat varieties and in early-infected wheat, rotten rhizomes, root death and stem distortion can also be observed. In our previous work, phylogenetic analysis based on *16S rRNA* and ribosomal protein gene sequences of WBD and other phytoplasmas indicated that WBD phytoplasma belonged to the 16Sr I group [11]. Recent research has shown the polymorphisms of WBD phytoplasma, three subgroups 16Sr IB, 16Sr IC and 16Sr IS were found in WBD phytoplasma [12]. In this paper, a WBD phytoplasma isolate belonging to the 16Sr IB subgroup was sequenced.

Whole genome amplification (WGA) is a technique to produce a large quantity of DNA from limited DNA source. Multiple displacement amplification (MDA) is one of the principal methods of WGA. It generates large fragments (>10 kb) with a low error frequency (one in 10^6^–10^7^) [13]. MDA-based WGA has significant implications for genetic studies, forensic medicine, clinical diagnostics and genome sequencing projects [14].

In the present study, the draft genome of WBD phytoplasma and the comparative analysis between the WBD phytoplasma draft genome and the complete phytoplasma genomes are reported. Our work will provide an overall understanding about the metabolism of WBD phytoplasma and will also lay the groundwork for exploring the interactions between WBD phytoplasma and its hosts. Meanwhile, our work may provide a new strategy for the genome sequencing of other phytoplasmas and obligate organisms.

## Materials and Methods

### Phytoplasma sources

Wheat seedlings infected with WBD phytoplasma were collected from winter wheat fields belonging to farmers in Hancheng, Shaanxi Province, China. After obtaining permission from the landowners, the infected wheat seedlings were collected for our further studies. WBD phytoplasma was transmitted to healthy periwinkle plants (*Catharanthus roseus*) from infected wheat seedlings by *Psammotettix striatus*. It was then maintained and propagated in an insect-proof greenhouse by periodic grafting. WBD phytoplasma infection in the periwinkle was confirmed by nested PCR using P1/P7 and R16F2n/R16R2 (Table S1) and the 16S rDNA sequence.

### WBD phytoplasma chromosomal DNA isolation

WBD phytoplasma chromosomal DNA isolation was conducted according to the procedure developed in our previous study [15]. Briefly, WBD phytoplasma pellets were purified by differential centrifugation and then subjected to pulsed-field gel electrophoresis (PFGE) after digestion by proteinase K at 50°C for 72 h. A band of 650 kb in the PFGE gel was identified as WBD phytoplasma chromosomal DNA by Southern blot analysis. The DNA was then electro-eluted to dialysis tubing.

### WGA by MDA

The electroeluate was first concentrated using Centrifugal Filter Units (Millipore, Germany). Concentrated DNA was used as a template for MDA. MDA was carried out using the REPLI-g Mini Kit (Qiagen, Germany) according to the manufacturer's instructions. The DNA was denatured in a denaturation buffer and then amplified in a 40-µL reaction overnight at 30°C. The reaction was terminated by a 3 min incubation at 65°C. The product was stored at −80°C for future use.

### Real-time PCR analysis

The primers RT16s999 and RT16s1162 were designed based on the WBD phytoplasma *16S rRNA* gene sequence to quantify WBD phytoplasma chromosomal DNA. The real-time PCR reactions were carried out using the SYBR premix Ex Taq II kit (Takara-Bio, Dalian, China) with the following PCR conditions: 95 °C for 1 min, 40 cycles of 95 °C for 10 s, 50 °C for 20 s, and 72 °C for 40 s. Melting curves were analyzed at the end of each amplification. All samples were tested in three biological replicates. Two microliters of the 10× WGA-generated DNA was used as the template for real-time PCR analysis, and an absolute standard curve was established using a series of 10-fold plasmid DNA cloned from PCR amplicons of the *16S rRNA* gene.

### Shotgun sequencing and genome assembly

Shotgun sequencing was conducted in the Genomics Core Facility of Purdue University, West Lafayette, USA. Instead of a pure DNA sample, the WGA product was used to prepare the sequencing libraries. Two libraries were prepared and sequenced. The CLC Genomic workbench (CLC-bio, Denmark) was run for *de novo* assembly. SSPACE-BASIC v2.0 [16] was used to scaffold the pre-assembled contigs. To screen the WBD phytoplasma contigs, the assembly was utilized as a series of queries to search against the complete phytoplasma genome sequence with TBLASTN [17]. Contigs homologous to phytoplasma complete genome sequences were subjected to further confirmation. Confirmation primers (Table S1) were designed based on the contig sequences, and a series of confirmation PCR reactions were carried out against both the WBD phytoplasma-infected and healthy periwinkle DNA to determine which of the contigs were indeed from WBD phytoplasma. Finally, outward primers (Table S1) were designed based on the sequences of PCR-confirmed contigs, and extensive PCR reactions were implemented to extend and join the WBD phytoplasma-associated contigs. All derived fragments were cloned into the pMD18-T simple vector (Takara-Bio, China) and sequenced.

### Genome annotation and analysis

Prodigal v2.60 [18] was used to predict open reading frames (ORFs) for the WBD phytoplasma genome sequence. UGA was used as a stop codon, which was consistent with the ORF prediction for other phytoplasmas. tRNAscan-SE was applied to identify tRNAs [19]. RNAmmer [20] was used to predict the locations of rRNA genes. WBD phytoplasma genes were named according to their homologous genes, which were identified by the OrthoMCL [21] and BLSTP [17] programs. OrthoMCL was used to identify homologous genes between the WBD phytoplasma genome and other complete phytoplasma genomes. Those genes with no homology to the other complete phytoplasma genomes were searched by BLASTP against the NCBI nr database. The rest of the genes, which had no homology with the phytoplasma complete genomes or the NCBI nr database, were presumed to be putative protein-coding genes only when they were longer than 100 amino acids or had a confidence score of more than 10 from Prodigal.

To understand the functions of genes from the WBD phytoplasma genome, KAAS [22] and COG [23] were used for gene functional categorization. First, the protein sequences of all genes were annotated by KAAS using a gene data set default that was representative of prokaryotes, Tenericutes and Firmicutes. Bi-directional best hit was used as the assignment method. Next, genes with a KEGG orthology assignment were further assigned to a COG functional category. The genes without any functional category assignment were put in a category named X.

To identify the putative secreted proteins, we referenced the methods described by Bai and colleagues [24]. Only those proteins that did not contain transmembrane (TM) regions after cleavage of the signal peptide (SP) were predicted to be putative secreted proteins. The program signalP V3.0 [25] was used to predict SP cleavage in all proteins. The organism group was set as Gram-positive bacteria, using the method of hidden Markov models. For SP-containing proteins, TMHMM V2.0 [26] was used to predict the transmembrane regions after eliminating the SP. For putative secreted proteins, PSORT [27] was further applied to predict their localization within plant and animal cells.

### Comparative analysis with other phytoplasma genomes

For comparative genomic analysis, two complete phytoplasma genomes (strains OY-M [GenBank accession number: AP006628] and AY-WB [GenBank accession number: CP000061]) from the 16Sr I group were chosen for comparison with the WBD genome. A 16Sr XII group phytoplasma ‘*Ca*. P. australiense’ (Subgroup tuf-Australia I; rp-A) [GenBank accession number: AM422018] and a 16Sr X group phytoplasma ‘*Ca*. P. mali’ [GenBank accession number: AM422018]) were also chosen for comparison. r2cat [28] was used to provide an interactive visualization for synteny inspection between the WBD genome and these complete genomes. To determine the placement of the WBD contigs in the genomes of OY-M and AY-WB, TBLASTN [17] was used to analyze the sequence similarity of the WBD contigs and these two genomes. To identify shared and genome-specific genes, OrthoMCL was applied to identify homologous genes between the WBD phytoplasma genome and other phytoplasma genomes. BLASTP was used for the alignment of protein sequences.

## Results and Discussion

### Concentration of WBD phytoplasma chromosomal DNA

A real-time PCR assay was carried out to quantify the concentration of the WBD phytoplasma chromosomal DNA in MDA production. According to the published phytoplasma genome, two sets of *16S rRNA* genes exist in one phytoplasma; the concentration of WBD chromosomal DNA (C) tends to vary linearly with the copy number of 16S rDNA (CN) in WGA production.
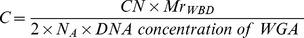



In our previous work [15], PFGE revealed that the WBD phytoplasma chromosome is approximately 650 kb. The copy number of the WBD phytoplasma in 10× diluted MDA-generated DNA was 2.49×10^7^/µL (Fig. S1), and the DNA concentration of WGA production was 94 ng/µL; therefore, the ratio of WBD phytoplasma chromosomal DNA was 92.9%. In addition to the WBD phytoplasma chromosomal DNA, a small amount of plant DNA existed in the WGA production, which was proven by the contigs assembled by the CLC Genomic Workbench. Several contigs with low coverage were homologous to sequences of plant chromosome DNA and chloroplast DNA. Nonetheless, after PFGE and several WGA reactions, the amount of high-quality WBD phytoplasma DNA was enough for sequencing. Until now, two methods were used for phytoplasma DNA purification in phytoplasma complete genome projects. One method was PFGE, and the other was repeated bisbenzimide-CsCl buoyant-density-gradient centrifugation. However, both methods were time-consuming processes to gather enough high-quality phytoplasma DNA for sequencing. To date, WGA using MDA has been successfully applied to many non-culturable bacteria sequencing projects, including an uncultivated SAR324 clade of Deltaproteobacteria [29] and Citrus Huanglongbing Bacterium ‘*Candidatus* Liberibacter asiaticus’ [30]. In the present study, using the DNA from PFGE as a template, the WGA technique was utilized to amplify WBD phytoplasma DNA. This not only assured the high purity of the WBD phytoplasma DNA but also greatly improved the efficiency of the WBD phytoplasma DNA collection. Using this approach, it took only one week to obtain enough high-quality WBD phytoplasma DNA from small amounts of WBD phytoplasma-infected periwinkle leaves. The method adopted in this work provides a new strategy for the collection of DNA from other phytoplasmas for genome sequencing.

### WBD phytoplasma genome assembly

Shotgun sequencing with Illumina HiSeq 2000 and MiSeq generated 9,980,498 read-pairs (insert size = ∼200 bp, 1.85 Gb of raw data) and 23,694,055 read-pairs (insert size = ∼500 bp, 9.27 Gb of raw data), respectively. These read-pairs were assembled by *de novo* assembly into 3,957 contigs covering 12,684,620 bp. The contigs ranged from 1000 to 274,305 bp. Pre-assembled contigs were extended into 3,902 contigs covering 12,713,367 bp. After performing TBLASTN searches against the complete phytoplasma genome sequences, 40 contigs (Table S2) covering 649,537 bp were found to be homologous to sequences within the complete phytoplasma genome. Next, PCR with WBD phytoplasma-infected and healthy periwinkle DNA (Fig. S2) confirmed that 16 contigs (Table S2) covering 604,448 bp were from WBD phytoplasma. These 16 contigs were further assembled by extensive PCR reactions using outward primers (Fig. S3). Finally, 6 contigs (Table S2) covering 611,462 bp were obtained. According to previous work, the WBD phytoplasma chromosome is approximately 650 kb in size; therefore, our assembly covered ∼94% of the WBD phytoplasma genome. Unlike in AY-WB and PnWB, no plasmid sequences were found in the WBD assembly. This may be because the WBD phytoplasma plasmid DNA was too low in concentration to be amplified by WGA. However, a region (from *WBD_0259* to *WBD_0264*) located in contig 3 was similar to that from WBD phytoplasma plasmids published in our previous work [31]. This phenomenon has been reported in the AY-WB genome, in which ORFs from three AY-WB chromosomal segments were similar to ORFs from AY-WB plasmids [4].

A large number of contigs were obtained by *de novo* assembly, making it challenging to screen phytoplasma-associated sequences. Fortunately, many phytoplasma sequences have been published, which could be used for the initial screening of phytoplasma-associated sequences. In this paper, TBLASTN searches were performed against the complete phytoplasma genome sequence, greatly reducing the number of confirmation PCR reactions and significantly increasing the efficiency of assembly.

Despite having more than 10 GB of raw data and running extensive PCR reactions, we were unable to assemble the complete genome. This may be due to host DNA contamination in the sequence samples and repetitive sequences in the WBD phytoplasma genome. Given that four of the five complete phytoplasma genomes are circular and that the genomes of OY-M and AY-WB, which belong to the same 16Sr group as WBD phytoplasma, are circular, the WBD phytoplasma genome was also presumed to be circular.

### General features of the WBD phytoplasma draft genome

The Whole Genome Shotgun project was uploaded to DDBJ/EMBL/GenBank under accession AVAO00000000. The version described in this paper is version AVAO01000000.

**Figure 1:**
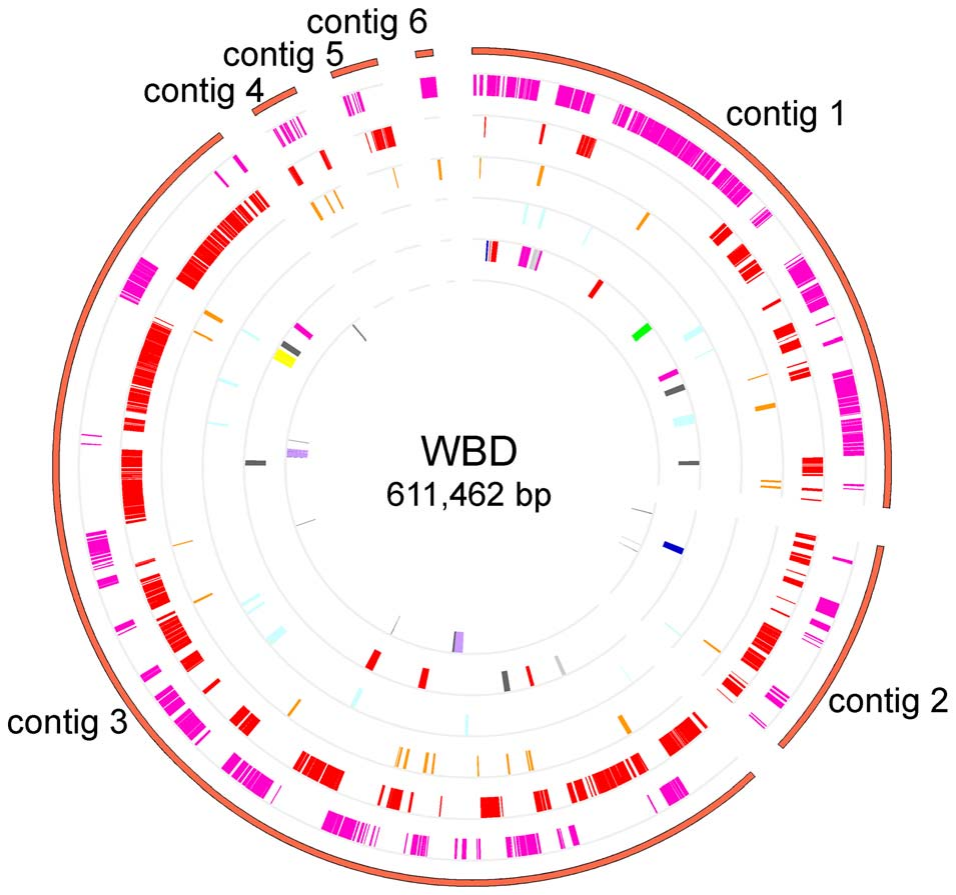
Map of the WBD draft genome. Rings from the outside to inside are as follows: ring 1, contigs of the WBD draft genome; ring 2, predicted ORFs on the sense strand; ring 3, predicted ORFs on the antisense strand; ring 4, ORFs of predicted secreted proteins; ring 5, ORFs of predicted secreted membrane proteins; ring 6, ORFs of predicted transport proteins; ring 7, locations of rRNA genes (purple) and tRNA genes (gray).

**Figure 2:**
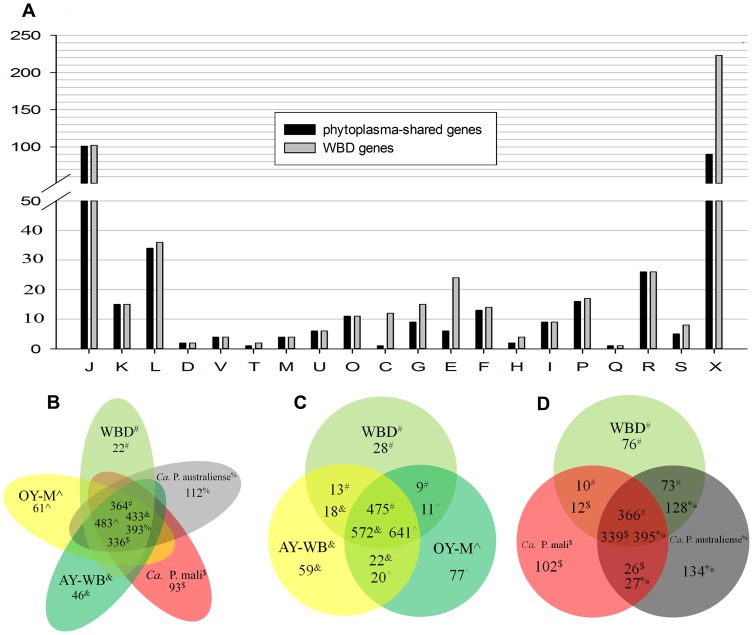
Number of homologous genes and genome-specific genes. A, comparative analysis of the COG functional category between WBD phytoplasma genes and phytoplasmas-shared genes; B, comparison among WBD, OY-M, AY-WB, ‘*Ca*. P. mali’ and ‘*Ca.* P. australiense’; C, comparison among WBD, OY-M and AY-WB; D, comparison among WBD, ‘*Ca*. P. mali’ and ‘*Ca.* P. australiense’. J: Translation, ribosomal structure and biogenesis; K: Transcription; L: Replication, recombination and repair; D: Cell cycle control, cell division, chromosome partitioning; V: Defense mechanisms; T: Signal transduction mechanisms; M: Cell wall/membrane/envelope biogenesis; U: Intracellular trafficking, secretion, and vesicular transport; O: Posttranslational modification, protein turnover, chaperones; C: Energy production and conversion; G: Carbohydrate transport and metabolism; E: Amino acid transport and metabolism; F: Nucleotide transport and metabolism; H: Coenzyme transport and metabolism; I: Lipid transport and metabolism; P: Inorganic ion transport and metabolism; Q: Secondary metabolite biosynthesis, transport and catabolism; R: General function prediction only; S: Function unknown; X: no functional category assignment.

The WBD phytoplasma genome is comprised of six contigs covering 611,462 bp, with a G+C content of 27.14% (Table 1, Fig. 1). Upon annotation, 525 protein-coding genes, two operons for rRNA genes and 32 tRNA genes were found within the genome. Of the 525 annotated protein-coding genes, 269 had specific functional assignments according to the COG categories. These 269 genes were assigned to 17 functional categories. The most abundant functional category was COG category J (translation and ribosomal structure), which accounted for approximately 20% of the WBD genes, mostly due to 51 ribosomal proteins and 21 tRNA synthetases found in the genome. The second most abundant was COG category L (replication, recombination and repair), which contained 36 genes (Fig. 2A).

**Table 1:** Genome assembly statistics.

Characteristic	Value for group				
	WBD	OY-M	AY-WB	‘ *Ca*. P. mali’	‘ *Ca*. P. australiense’
No. of contigs	6	1	1	1	1
Length (bp),	611, 462	853, 092	706, 569	601, 943	879, 959
G+C content (%),	27.1	27.8	26.9	21.4	27.4
No. of protein-coding genes	530	749	671	479	684
Coding density (%),	71.6	72.8	73.5	76.1	64.1
No. of tRNA genes	32	32	31	32	35
No. of rRNA operons	2	2	2	2	2
No. of plasmid	3	2	4	0	3

Because the complete WBD genome was not obtained, sequences of six contigs were used to predict the putative origin of replication (oriC) by Oriloc [32]. The result of Oriloc indicated that the WBD genome has an irregular GC-skew (Fig. S4); the position of the oriC was not clear. In OY-M and AY-WB, the first nucleotide of the *dnaA* sequence was defined as bp 1. Consequently, the first nucleotide of *dnaA* was defined as bp 1 of the WBD phytoplasma genome, which is located at 138217 bp in contig 1.

### Comparative genomic analysis of phytoplasmas

**Figure 3:**
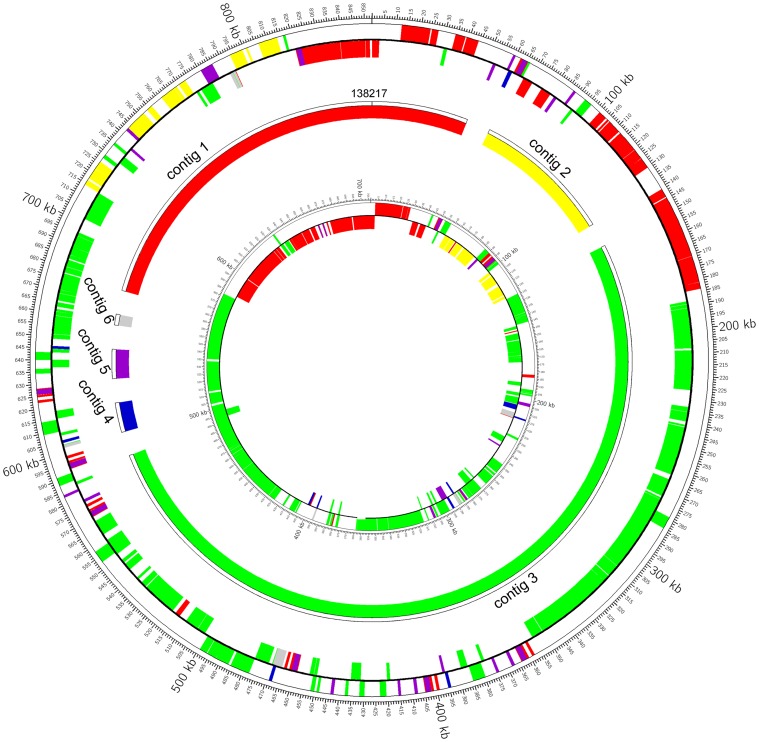
Placement of WBD contigs in the OY-M and AY-WB genomes. Ring 1, similar sequences to the WBD contigs in the OY-M sense strand; ring 2, similar sequences to the WBD contigs in the OY-M antisense strand; ring 3, WBD contigs; ring 4, similar sequences to the WBD contigs in the AY-WB sense strand; ring 4, similar sequences to the WBD contigs in the AY-WB antisense strand.

The placement of the WBD contigs in OY-M and AY-WB was analyzed by TBLASTN. The similar sequences of WBD contigs were positioned in OY-M and AY-WB by different colors (Fig. 3). Each WBD contig was distributed throughout the OY-M and AY-WB genomes. Similar sequences of some contigs, such as contig 1 and contig 3, were spread across two strands of the OY-M and AY-WB genomes. These revealed that extensive rearrangements and inversions existed in these three genomes (Fig. 3). Because the WBD phytoplasma genome is comprised of six contigs, only genomic rearrangements and inversions that occurred within contigs are identifiable. Thus, the actual number of rearrangements and inversions may be higher.

**Figure 4:**
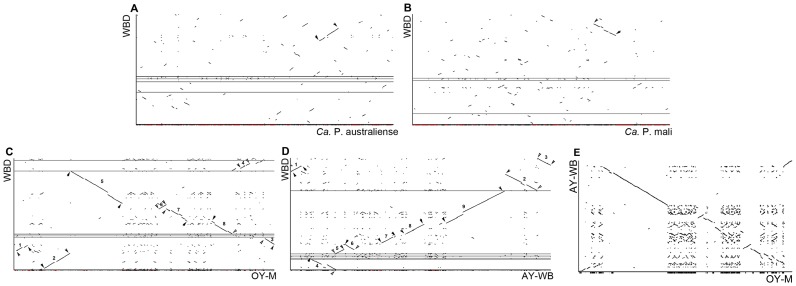
Synteny plots of WBD and other phytoplasma genomes. Shown are alignments of the WBD assembly and ‘*Ca.* P. australiense’ (A), ‘*Ca.* P. mali’(B), OY-M (C) and AY-WB (D). E, alignment between OY-M and AY-WB.

Genomic synteny analysis between the WBD phytoplasma genome and other complete phytoplasma genomes were performed using r2cat. The alignment among the genomes of WBD phytoplasma, ‘*Ca*. P. australiense’ and ‘*Ca*. P. mali’ showed a few regions of synteny (Fig. 4A, Fig. 4B). The conserved region was ∼68 kb between WBD phytoplasma and ‘*Ca*. P. australiense’ and ∼62 kb between WBD phytoplasma and ‘*Ca*. P. mali’. However, the alignments between the genomes of WBD, OY-M and AY-WB displayed more regions of synteny (Fig. 4C, Fig. 4D). This illustrates that WBD has significant genomic synteny with OY-M and AY-WB. Eight conserved regions covering ∼444 kb were found between WBD and OY-M. The largest (∼175 kb) of these was located in contig 3 and was in the reverse orientation. Nine conserved regions covered ∼494 kb between WBD and AY-WB, and the largest alignment region was ∼176 kb. This region was also located in contig 3 but had the same orientation. The details of these conserved regions are shown in Table S3.

To compare the gene content of the WBD phytoplasma genome with the complete genomes of other phytoplasmas in the same and different 16Sr groups, homologous genes were identified among these genomes. A large number of homologous genes were identified: 364 from WBD, 483 from OY-M, 433 from AY-WB, 336 from ‘*Ca*. P. mail’ and 393 from ‘*Ca*. P. australiense’ (Fig. 2B). The presence of homologous genes in all five genomes revealed their importance in phytoplasma. Of the 364 homologous genes of WBD phytoplasma, 244 had specific functional assignments; the remaining genes were without functional assignments. Almost all of the WBD phytoplasma genes with specific functional assignments were homologous to genes of other phytoplasmas, except for several genes involved in energy production and conversion (COG category C), carbohydrate transport and metabolism (COG category G), and amino acid transport and metabolism (COG category E). However, homologous genes without functional assignments accounted for 40.7% of the total non-functional genes in the WBD phytoplasma genome (Fig. 2A). When comparing WBD phytoplasma with the other two 16Sr I group phytoplasmas, OY-M and AY-WB, a few more homologous genes were found: 475 from WBD, 572 from AY-WB and 641 from OY-M (Fig. 2C). WBD phytoplasma and the other two 16Sr group phytoplasmas ‘*Ca*. P. australiense’ and ‘*Ca*. P. mail’ shared fewer homologous genes: 366 from WBD, 395 from ‘*Ca*. P. australiense’ and 339 from ‘*Ca*. P. mail’ (Fig. 2D). These results illustrated that more similar genes were found in different strains of the same 16Sr group phytoplasmas. In addition to these homologous genes, WBD phytoplasma contained 22 specific genes. However, the majority of these WBD-specific genes had no assigned functions. Only one gene had an assigned function and was present in two copies. This gene encoded a protein with the highest amino acid identity with a hydrolase of *Staphylococcus epidermidis*. The phytoplasma genomes used for this comparison were complete sequences, and all genes of these phytoplasmas were obtained. In contrast, the WBD phytoplasma genome was a draft genome; therefore, some WBD phytoplasma genes may have been lost. Thus, there may have been more homologous genes and WBD-specific genes.

**Figure 5:**
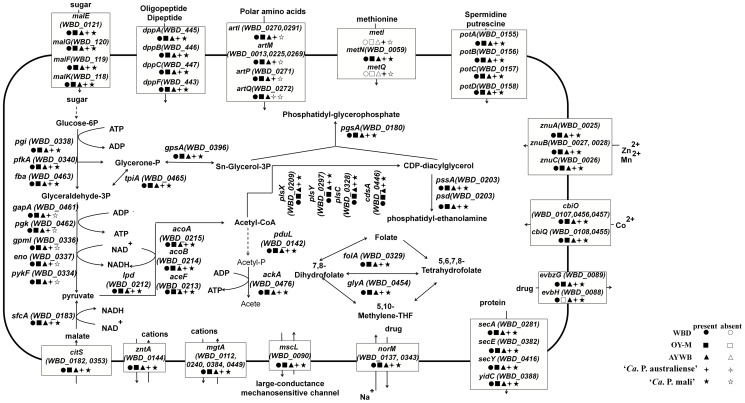
Summary of the genome-encoded transporters and central metabolic pathways of WBD and other phytoplasma. The dotted line represents a lack of genes encoding an enzyme involved in the reaction.

**Figure 6:**
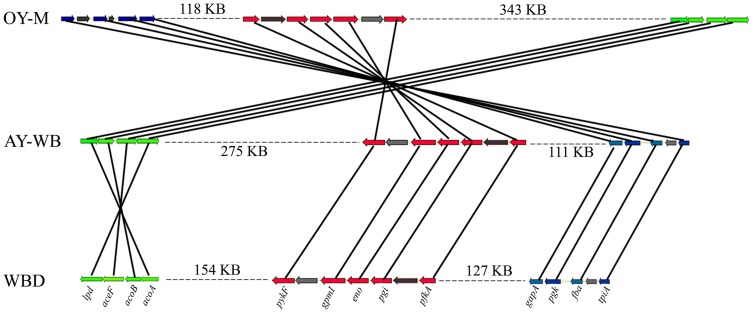
The arrangements of 13 genes involved in glycolysis and gluconeogenesis in the WBD, OY-M and AY-WB genomes.

To study the metabolites of WBD phytoplasma, pathways were predicted based on the protein sequences of the WBD genome using the KAAS tool. The results are summarized in Fig. 5. As is the case in other phytoplasmas, many pathways cannot be found in the WBD phytoplasma genome, indicating that its metabolic capabilities are highly reduced. The complete pathways for amino acid and fatty acid biosynthesis, oxidative phosphorylation, tricarboxylic acid cycle and pentose phosphate pathway cannot be found in the WBD phytoplasma genome. Furthermore, none of the ATP-synthase subunits were identified in the WBD phytoplasma genome, suggesting that WBD phytoplasma either depends on glycolysis for its energy generation or imports ATP from its host. However, the mechanism required to import ATP from its host is still unclear, glycolysis has been observed in phytoplasma. Thirteen proteins involved in glycolysis and gluconeogenesis are encoded by the WBD phytoplasma genome. The amino acid sequences of these 13 proteins are 16Sr group specific. When comparing these proteins between WBD phytoplasma and ‘*Ca*. P. mali’ and ‘*Ca*. P. australiense’, the amino acid sequence identity is not more than 85.8%. However, the sequence identity ranges from 89.2% to 97.9% when comparing WBD phytoplasma with different strains of the same 16Sr group, AY-WB and OY-M (Table 2). Furthermore, in WBD, OY-M and AY-WB, these 13 genes are all located in three regions of the genomes with different orders. Relative to the WBD phytoplasma genome, all 13 genes in OY-M and four genes (*lpd*, *aceF*, *acoB* and *acoA*) in AY-WB are in the reverse orientation (Fig. 6). Although 13 genes involved in glycolysis were found in the WBD phytoplasma genome, phosphoenol pyruvate dependent sugar phosphotransferase (PTS) systems, which are responsible for sugar importation and phosphorylation, were found to be absent. Instead, four proteins for the ABC transporter of maltose (MalKFGE) were found in WBD phytoplasma (Fig. 5). It has been reported that the maltose ABC transporter could also recognize trehalose, sucrose and palatinose in some bacteria [33]. Sucrose and trehalose are major sugars in plant phloem and insect hemolymph, respectively. However, the enzymes that use these sugars as substrates to produce α-D-Glucose-6P were not identified in the WBD phytoplasma genome. In addition to sugar, malate can also be utilized as a carbon source for ATP production in WBD phytoplasma [7]. Furthermore, sequences coding for a protein that transports malate and citrate and an enzyme that uses malate as a substrate to produce pyruvate were identified in the WBD phytoplasma genome. Moreover, four enzymes involved in the pyruvate to acetyl-CoA pathway and an enzyme using acetyl-p and ADP as a substrate to produce acetate and ATP were encoded by the WBD phytoplasma genome. Although phosphate acetyltransferase, which uses acetyl-CoA as a substrate to produce acetyl-p, was not found, phosphate propanoyltransferase was identified, which could catalyze this reaction in phytoplasma. Compared with sugar, malate may play a more important role in the ATP supply of phytoplasmas because enzymes for the ATP-yielding reactions of glycolysis were not found in ‘*Ca*. P. mali’ (Fig. 5) [7].

**Table 2:** Amino acid sequence identity of 13 proteins involved in glycolysis and gluconeogenesis between WBD and four other phytoplasmas.

	OY-M	AY-WB	‘*Ca*. P. mali’	‘*Ca*. P. australiense’
*pykF*	95.1%	92.4%	-	56.1%
*gpmI*	94.5%	94.5%	-	68.6%
*eno*	96.5%	95.1%	-	80.0%
*pgi*	95.5%	94.1%	54.2%	72.8%
*pfkA*	93.0%	92.1%	44.8%	78.3%
*lpd*	94.5%	93.7%	65.6%	72.4%
*aceF*	90.1%	89.2%	50.8%	71.3%
*gapA*	97.9%	97.0%	-	84.8%
*pgk*	92.0%	93.5%	-	72.9%
*fba*	97.6%	94.8%	62.4%	77.4%
*tpiA*	94.1%	93.4%	50.4%	62.3%
*acoB*	96.9%	96.3%	69.8%	85.8%
*acoA*	97.0%	95.9%	61.4%	82.4%

As is the case in other phytoplasmas, the pathways for ATP synthesis and fatty acid metabolism in WBD phytoplasma are incomplete. Only one gene involved in oxidative phosphorylation (inorganic pyrophosphatase) and eight genes related to glycerolipid and glycerophospholipid metabolism were identified.

The WBD phytoplasma genome also lacks genes involved in the synthesis of several essential amino acids, although it does have eight genes involved in the metabolism of alanine, aspartate, glutamate, glycine, serine, threonine, valine, leucine, isoleucine and lysine. The WBD phytoplasma genome encodes eleven genes involved in thiamine, nicotinate and nicotinamide, pantothenate and CoA, lipoic acid and folate metabolism. However, it lacks a vitamin B_6_ metabolism gene, which is found in the OY-M phytoplasma genome. Four genes (*folK*, *folP*, *folA* and *folC*) involved in folate biosynthesis exist in the OY-M genome. However, the *folC* gene is absent in WBD phytoplasma.

Phytoplasmas have strongly reduced metabolic capabilities; therefore, they must absorb basic metabolites from their plant and insect hosts. Forty-six genes encoding transport and binding proteins were identified in the WBD phytoplasma genome (Fig. 5, Table S4), which is a large number of transporter systems relative to its genome size. These include ATP-binding cassette (ABC) transporter systems for molecules such as dipeptides/oligopeptides, spermidine/putrescine, cobalt and Mn/Zn. The components of each ABC transporter are clustered in the WBD phytoplasma genome, which is consistent with observations in the five complete phytoplasma genomes. The methionine permease component was not found in WBD phytoplasma, but there was a gene (*WBD0061*) that was similar to a D-methionine transport system permease protein of ‘*Ca*. P. australia’. Similar to the genomes of AY-WB and ‘*Ca*. P, mali’, the WBD phytoplasma genome contains two ABC transporters (EvbHG) involved in multidrug resistance. In addition to these transporters, WBD phytoplasma also encodes some unclassified transporters and other transporters, including cation transport ATPases and five P-type ATPases (4 *MgtA*, 1 *ZntA*). Similar to other phytoplasmas, WBD phytoplasma can also import malate and citrate. Two copies of *citS* involved in malate and citrate import are encoded in the WBD phytoplasma genome.

**Figure 7:**
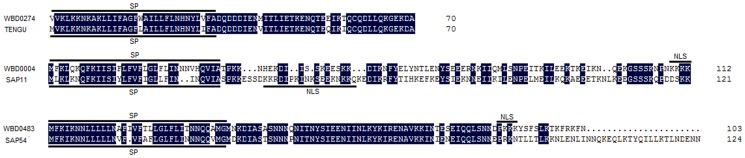
Alignments between WBD phytoplasma secreted proteins and published phytoplasma virulence factors.

Six genes involved in the bacterial secretion system were identified in the WBD phytoplasma genome (Fig. 5). These genes encode secA, secE and secY proteins, which are the essential components of the sec system [34]. Their presence suggests that a functional sec-dependent system exists in WBD phytoplasma. Proteins secreted via the sec-dependent pathway have N-terminal SP sequences. Fifty proteins encoded in WBD phytoplasma chromosomal DNA have predicted SP sequences. Of the 50 proteins, 18 contain predicted transmembrane regions in addition to SP sequences that are likely to remain attached to the WBD phytoplasma membrane after secretion. The remaining 32 proteins, which do not have extra transmembrane regions after removing their SP sequences, were identified as secreted proteins. In addition to these 32 secreted proteins (Table S5), 5 secreted proteins were encoded by three WBD phytoplasma plasmids. With the exception of 9 proteins, these secreted proteins had no assigned functions. Of the 9 proteins with assigned functions, one protein was identified as CTP synthetase, three proteins encoded by plasmids were identified as copy number control proteins, and five proteins were identified as solute binding ABC transporters involved in sugar, Mn, methionine, dipeptide and polar amino acid transport. Of the remaining 28 unassigned-function proteins, only one putative secreted protein encoded by *WBD_0439* was a WBD phytoplasma-specific protein; the other 27 proteins were homologous with the proteins encoded by the other phytoplasmas. The virulence factor TENGU [35] from OY-M phytoplasma, and effectors SAP11 [36] and SAP54 [37] from AY-WB phytoplasma, have been shown to alter plant morphology. The protein encoded by *WBD_0274* had a 91.43% amino acid sequence similarity with the TENGU factor from OY-M phytoplasma. Two proteins encoded by *WBD0004* and *WBD0483* were similar to the two AY-WB effectors SAP11 and SAP54 (with E-values of 1e-40 and 7e-42, respectively) (Fig. 7). Given that WBD phytoplasma-infected periwinkle displays shoot proliferation, virescence and phyllody, these three secreted proteins may be responsible for such abnormalities. Of these three proteins, putative nuclear localization signals (NLSs) were found in the proteins encoded by *WBD0004* and *WBD0483*. In addition to these two proteins, NLSs were also found in two additional proteins encoded by *WBD0235* and *WBD0253*. These four proteins may target the plant cell nucleus after their secretion. Of the four putative nuclear-targeted proteins, only the protein encoded by *WBD_0253* had a bipartite NLS; the other proteins had only monopartite NLSs.

Because phytoplasmas strictly live in plant phloem and insect hosts, they must adapt to diverse environments. Several proteins with similar functions were encoded in the WBD phytoplasma genome, including the two molecular chaperones DnaK and DnaJ. These two genes have been reported to be necessary for pathogen survival in the host cell, and knocking out the *dnaK*-*dnaJ* operon in *Salmonella enterica serovar Typhimurium* resulted in a mutant that could grow in culture but not *in vivo* and was unable to colonize mice [38]. In addition, a cold shock protein encoded by *cspC* and an hsp20 family protein encoded by *ibpA* may help WBD phytoplasma respond to several stresses. It has been reported that arsenate salt and some antibiotics, such as ampicillin, rifampicin and tetracycline, can induce the *cspC* promoter [39]. Hsp20 was induced in response to severe heat shock regimens and by osmotic shock in *Bifidobacterium breve* UCC2003 [40]. In addition to the hsp20 protein, the large-conductance mechanosensitive channel (MscL) protein, which may help WBD phytoplasma adapt to changes in the osmotic pressure between plant and insect cells, was identified. MscL has been found in bacteria, archaea, fungi and higher plants, where it is involved in the defense against osmotic shock [41]. In addition, a Mn-type SOD protein was encoded by the *sodA* gene in the WBD phytoplasma genome. This protein may play an important role in the degradation of reactive oxygen species (ROS) produced in the plant during phytoplasma infection [42]. The identification of these proteins suggests that WBD phytoplasma has several mechanisms that allow it to adapt to complex environments.

## Supporting Information

Figure S1 **Real-time PCR quantification of WBD phytoplasma chromosomal DNA in WGA production.**
(TIFF)

Figure S2 **PCR confirmation of WBD-associated contigs in WBD phytoplasma-infected and healthy periwinkle DNA.** Lane M, DM2000 plus (CWBIO, China): 5000 bp, 3000 bp, 2000 bp, 1000 bp, 750 bp, 500 bp, 250 bp, 100 bp; lane 1, healthy periwinkle; lane 2, WBD phytoplasma-infected periwinkle. White arrows point to the bands. The numbers above the PCR reactions correspond to the confirmed contigs.
(TIFF)

Figure S3 **PCR connection of the WBD-associated contigs.** Lane M, DS2000 plus (CWBIO, China): 5000 bp, 3000 bp, 2000 bp, 1000 bp, 750 bp, 500 bp, 250 bp, 100 bp; lane 1, healthy periwinkle; lane 2, WBD phytoplasma-infected periwinkle. White arrows point to the bands. The numbers above the PCR reactions correspond to the assembled contigs.
(TIFF)

Figure S4 **Cumulated skew of the WBD genome.**
(TIFF)

Table S1 **Primers used in this study.**
(XLS)

Table S2 **Description of the contigs in each stage of assembly.**
(XLS)

Table S3 **Details of conserved regions between WBD phytoplasma and other phytoplasmas.**
(XLS)

Table S4 **Transporters in the WBD phytoplasma and other phytoplasma complete genomes.**
(XLS)

Table S5 **Name and description of protein-coding genes in WBD phytoplasma.**
(XLS)
