# Supplementary figures and images for: Comparative Genome Analysis of Wheat Blue Dwarf Phytoplasma, an Obligate Pathogen That Causes Wheat Blue Dwarf Disease in China
Source: PLoS One. 2014 May 5;9(5):e96436. doi: 10.1371/journal.pone.0096436 (PMC4010473; doi:10.1371/journal.pone.0096436)

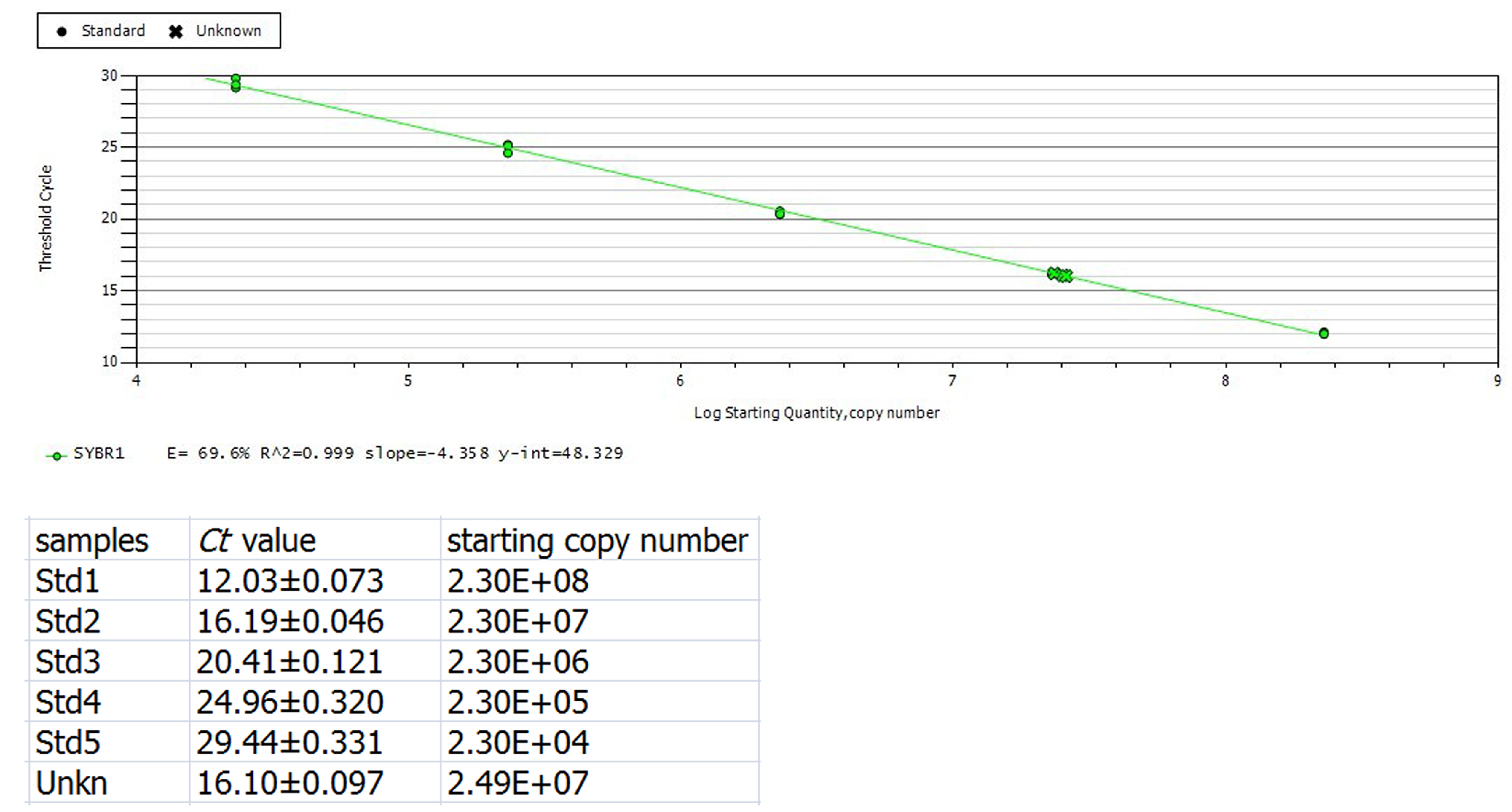

Supplement: Figure S1 — Real-time PCR quantification of WBD phytoplasma chromosomal DNA in WGA production. (TIFF) [file pone.0096436.s001.tiff]

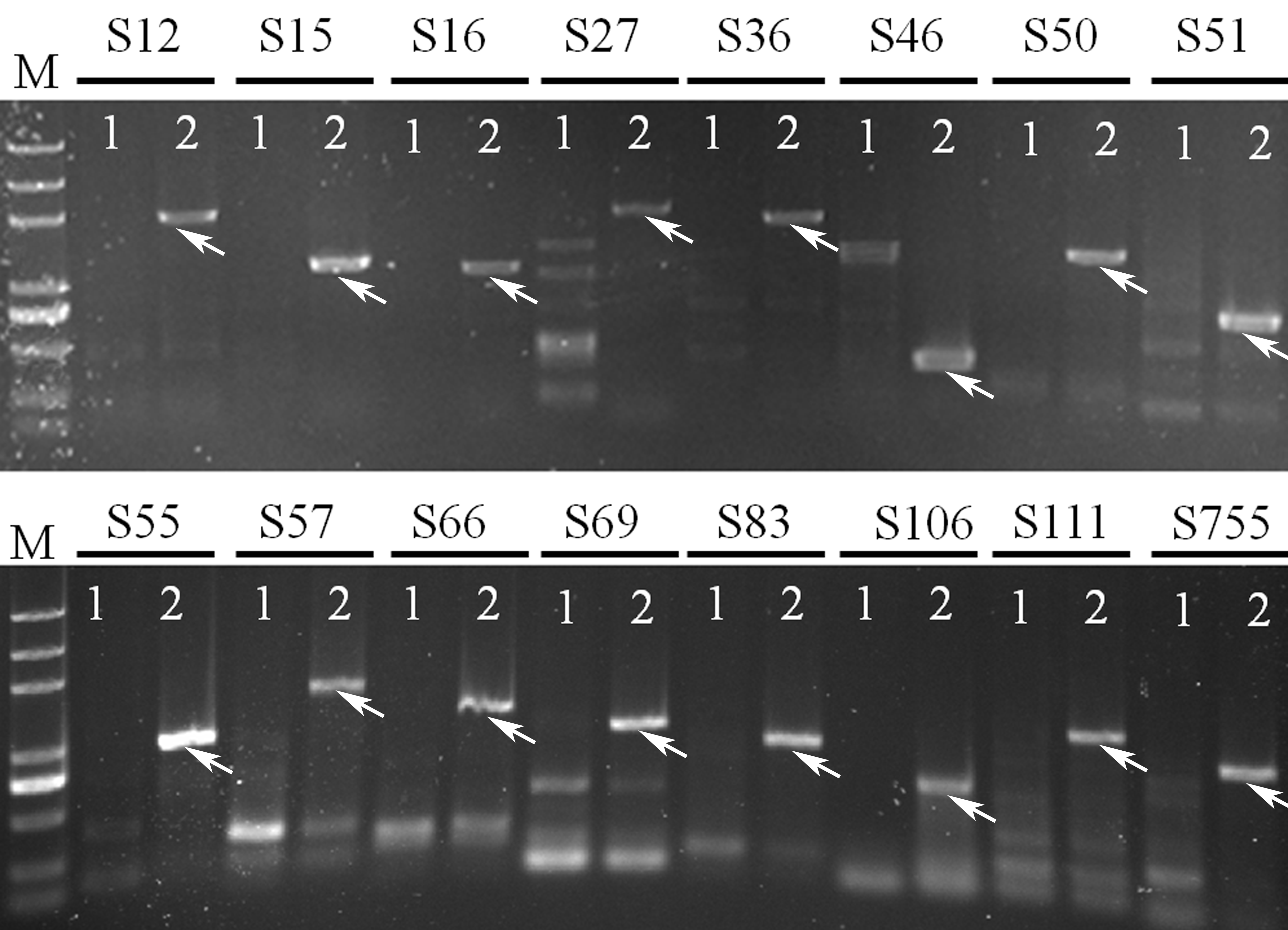

Supplement: Figure S2 — PCR confirmation of WBD-associated contigs in WBD phytoplasma-infected and healthy periwinkle DNA. Lane M, DM2000 plus (CWBIO, China): 5000 bp, 3000 bp, 2000 bp, 1000 bp, 750 bp, 500 bp, 250 bp, 100 bp; lane 1, healthy periwinkle; lane 2, WBD phytoplasma-infected periwinkle. White arrows point to the bands. The numbers above the PCR reactions correspond to the confirmed contigs. (TIFF) [file pone.0096436.s002.tiff]

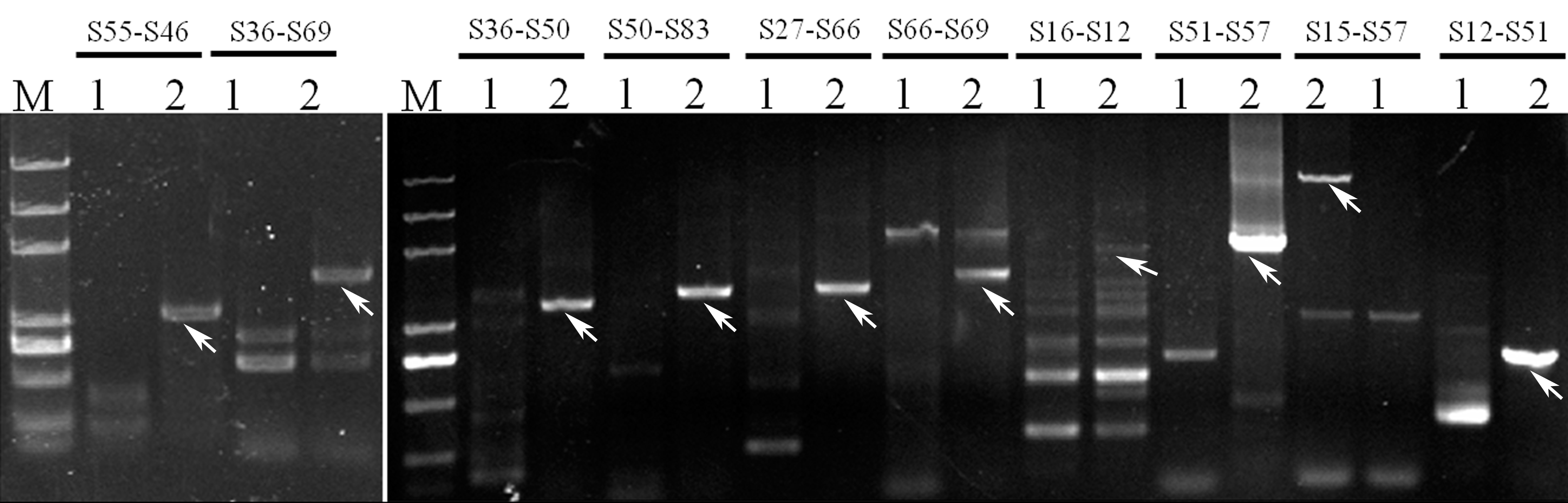

Supplement: Figure S3 — PCR connection of the WBD-associated contigs. Lane M, DS2000 plus (CWBIO, China): 5000 bp, 3000 bp, 2000 bp, 1000 bp, 750 bp, 500 bp, 250 bp, 100 bp; lane 1, healthy periwinkle; lane 2, WBD phytoplasma-infected periwinkle. White arrows point to the bands. The numbers above the PCR reactions correspond to the assembled contigs. (TIFF) [file pone.0096436.s003.tiff]

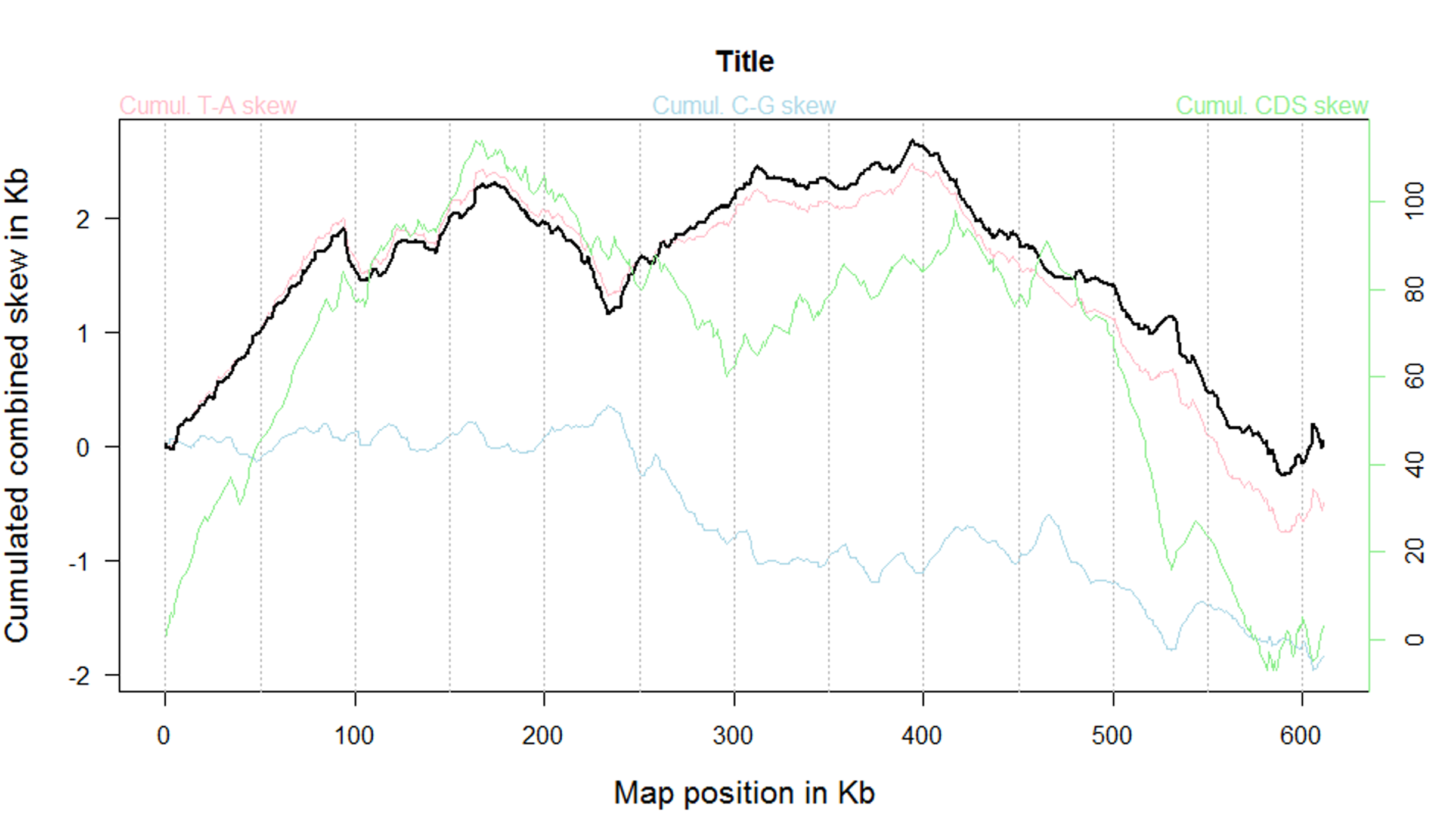

Supplement: Figure S4 — Cumulated skew of the WBD genome. (TIFF) [file pone.0096436.s004.tiff]
